# The associations of personality traits and parental education with smoking behaviour among adolescents

**DOI:** 10.1371/journal.pone.0174211

**Published:** 2017-03-23

**Authors:** Aina M. Yáñez, Alfonso Leiva, Andreu Estela, Iva Čukić

**Affiliations:** 1 Instituto de Investigación Sanitaria de Palma (IdISPa), Mallorca, Spain; 2 Research Group on Evidence, Lifestyles and Health, Universitat Illes Balears, Mallorca, Spain; 3 Primary Care Research Unit of Mallorca, Baleares Health Services-IbSalut, Mallorca, Spain; 4 Dalt Sant Joan Health Centre, Baleares Health services-IbSalut, Menorca, Spain; 5 Centre for Cognitive Ageing and Cognitive Epidemiology, University of Edinburgh, Edinburgh, United Kingdom; 6 Department of Psychology, University of Edinburgh, Edinburgh, United Kingdom; University of Granada, SPAIN

## Abstract

We examined whether personality traits and parental education are associated with smoking initiation in a sample of Spanish secondary school students. Participants, taken from the ITACA study (842 adolescents aged 14–15 years), completed a questionnaire assessing personality traits of the Five Factor Model, smoking behaviours and parental education. Multinomial logistic regression models controlling for age and sex were used to determine the independent associations and interactions of personality traits and parental education with risk of ever trying smoking, as well as with being a regular smoker in adolescence. Higher conscientiousness was related to a lower chance of trying smoking at least once (OR = 0.57, 95% CIs = 0.46, 0.71) as well as being a regular smoker (OR = 0.39, 95% CIs = 0.27, 0.55). Higher emotional instability (neuroticism) was associated with higher risk of being in either smoking category (OR = 1.33, 95% CIs = 1.10, 1.60 and OR = 1.76, 95% CIs = 1.31, 2.35, respectively). Higher extraversion was also associated with a higher risk of both types of smoking behaviour (OR = 1.38, 95% CIs = 1.12, 1.70 and OR = 2.43 (1.67, 3.55, respectively). Higher parental education was significantly related to lower risk of being a regular smoker (OR = 0.70, 95% CIs = 0.54, 0.89), but not with trying smoking in the past. Finally, we found no evidence of the interactions between adolescents’ personality and parental education in predicting adolescent smoking behaviours. We conclude that personality factors and parental education are important and independent factors associated with smoking behaviour in adolescents.

## Introduction

Smoking is one of the leading preventable causes of early death, disease and disability [1–3]. In addition, smoking is a major contributor to socio-economic inequalities in health since smoking-attributed mortality accounts for more than a half of the difference in mortality rates between social strata in middle age [4]. Smoking is typically taken up in adolescence [5], and early nicotine exposure directly increases risks of later nicotine dependence [6]. Given that the major risk period for smoking initiation is mostly over by the age of twenty [5, 7], understanding how environmental and individual risk factors contribute to smoking initiation in adolescence is a crucial step in designing appropriate intervention and prevention strategies.

Among environmental factors, socioeconomic status (SES), a combination of factors including income, education and occupation, has been shown to impact health due to lifestyle choices [8]. Furthermore, SES has been associated with adult smoking. For example, smoking is more common among people of lower education and lower SES levels in European countries [9, 10]. Some studies found an association between parental SES and smoking initiation of adolescents [11–13]. A recent cross-sectional study of 35 countries showed socioeconomic inequalities in adolescent smoking behaviour: boys and girls from poorer families were more likely to be smokers, and this association was mediated by an unequal distribution of family factors such as family structure and relationships with parents [12]. Crucially, while smoking rates among adults and adolescents are in decline in many western countries, including Spain [14, 15], these changes do not occur equally across different socioeconomic levels [10, 16, 17]. Adults with at least some college education had a significantly greater decline in smoking prevalence than those whose highest level of education is high school or less [18].

Among individual factors, personality traits described by the Five Factor Model (FFM)[19, 20], have been associated with a variety of health behaviours [21–23], including smoking [24, 25]. A study conducted on an elderly population showed that current smokers had higher levels of neuroticism, and lower levels of agreeableness and conscientiousness than former smokers, and those who never smoked had lowest levels of neuroticism and highest levels of agreeableness and conscientiousness of all groups [26]. Higher openness and higher neuroticism have been associated with lifetime-smoking, and higher conscientiousness appears to protect against smoking progression and persistence in adults [27]. A study conducted in an undergraduate student population showed that higher extraversion, higher neuroticism, lower agreeableness and lower conscientiousness were all associated with higher current smoking behaviour, and that higher neuroticism was more strongly associated with smoking in a Mexican, than in the Mexican-American sample [28]. Furthermore, Conner et al. [24] found that adolescent non-smokers had higher levels of conscientiousness than adolescent smokers.

Finally, Chapman et al. [25] evaluated, in an adult sample, whether the relationships between the level of education, a common marker of SES, and smoking behaviour would be confounded or modified by personality traits. While they found that never smokers had lower levels of openness and higher levels of conscientiousness, and that those who quit smoking had higher levels of neuroticism, they found that the effects of education and personality were additive, and therefore these two sets of risk factors should be considered independent.

To the best of our knowledge, the role of SES on the associations between personality traits and smoking in adolescents has not been explored to date. Building on the findings that parental SES affects smoking initiation among adolescents [12] and the studies that show that personality traits are associated with adolescent smoking status [24], here we explore whether personality traits and parental education are associated with smoking initiation in a cohort of Spanish secondary school students. Furthermore, we test whether the associations between personality traits and smoking vary across levels of parental education.

## Methods

### Sample

The study participants were students aged 14 to 15 years, who participated in the project ITACA: a multi-centre, cluster-randomized controlled trial, aimed at reducing the prevalence of smoking among secondary education students [29]. The initial ITACA sample comprised 1708 students (11–12 year-old) of 16 secondary education schools covering a wide range of communities (urban, semi urban and rural), socioeconomic status and prevalence of smoking. Schools were randomly assigned to a 4-year curriculum based multifactorial intervention or control groups. The research protocol was approved by the Primary Care Research Committee and the Balearic Ethical Committee of Research (IB 1146/09 PI). Here we focused on the second wave of assessment (October—December 2014) when personality was assessed. Participants met the inclusion criteria if they attended school on the day of the survey and if their parents agreed with participation in the study. The final sample comprised of 842 students (45.5% boys and 54.5% girls).

### Measures

#### Smoking status

Smoking status was assessed using seven items adapted from a previously validated questionnaire designed to assess smoking behaviours in adolescents [29]. Information on tobacco use was collected through the following question: “Which of the following statements best describes you? (A) I have never tried to smoke; (B) I have tried cigarettes a few times, but I do not smoke now; (C) I currently smoke at least one cigarette per month, but less than one cigarette per week; (D) I currently smoke at least one cigarette per week; (E) I smoke every day; (F) I used to smoke regularly in the past, but I do not smoke now”. The smoking status of adolescents were classified into never smokers (those who answered A) triers (answers B and F) and regular smokers (categories C, D and E).

#### Personality traits

Personality was assessed using the Big Five Questionnaire for Children (BFQ-C) [30] designed to assess the five basic personality traits: extraversion, agreeableness, conscientiousness, openness and emotional instability (neuroticism). Extraversion assesses characteristics such as activity, enthusiasm, assertiveness and self-confidence. Agreeableness assesses concern and sensitivity towards others and their needs. Conscientiousness assesses dependability, orderliness, precision, and fulfilling of commitments. Emotional instability refers to feelings of anxiety, depression, discontent, and anger and finally. Finally, openness taps both self-reported intellect, especially in the school domain, and broadness or narrowness of cultural interests and fantasy/creativity [30]. The Spanish adaptation of the questionnaire [31] included all 65 items with five possible responses, ranging from 5 (nearly always) to 1 (almost never). Reliabilities assessed by Cronbach’s Alpha in the present study were satisfactory: 0.87 for conscientiousness; 0.77 for extraversion; 0.82 for openness; 0.77 for instability; and 0.71 for agreeableness. Previous studies showed good psychometric properties of the BFQ-C [32, 33].

#### Parental education

The parental education measure describes the highest educational attainment of parents. The categories were as follows: a) incomplete primary education (less than six years of school), b) primary education completed (six-eight years), c) secondary education (four to six years), or d) university degree. The highest parental education variable was computed by taking the highest educational level obtained by either parent.

### Procedure

Students completed surveys during a 45-minute class at Grade 3 of secondary education. The surveys were administered by two trained data collectors. The teachers were asked to leave the classroom during the process to ensure students’ confidentiality. Written informed consent was obtained from all students and from at least one parent/guardian per student prior to the survey.

### Analyses

#### Descriptive statistics

To test differences in mean levels of personality traits between smoking categories a univariate analysis of variance (ANOVA) was calculated for the five personality traits. Similarly, to test whether parental educational levels differ across the smoking status categories, a Chi squared test was performed. The Pearson coefficient correlation was used to analyse the correlation between predictor factors.

#### Main analysis

Three multinomial logistic regression models were fitted to determine the independent associations of personality traits: extraversion, agreeableness, conscientiousness, emotional instability and openness, and parental education with smoking status. Odds ratios (ORs) were calculated for personality z-scores (SD = 1). The dependent variable was smoking status categorized as never smokers, triers and regular smokers. All models controlled for age, gender and allocation group (control and intervention). An overall model to test the statistical interaction between all possible interactions between personality traits and parental education was also fitted. All analyses were conducted using Stata version 11.0 (StataCorp, College Station, TX).

## Results

Descriptive statistics of the sample are presented in Table 1. Of the 842 adolescents included in the study, 62 (12.9%) self-reported being a smoker. Further 182 (21.6%) were classified as triers, and the remaining 598 (71%) as never smokers. Both triers and regular smokers were older than never smokers. Mean levels of extraversion and neuroticism were significantly higher among regular smokers and triers, and the mean level of conscientiousness was significantly lower among triers and regular smokers than in never smokers. There were significantly fewer smokers among adolescents whose parents had a university degree.

**Table 1:** Sample characteristics by smoking status.

	Total Sample	Never smokers	Triers	Smokers	*p*-value*
	N = 842	N = 598	N = 182	N = 62	
Age, mean (SD)	14.6 (0.6)	14.5 (0.5)	14.7 (0.6)	14.7 (0.6)	<0.001
Female n (%)	459 (54.5)	324 (54.2)	96 (52.7)	39 (62.9)	0.635
Male n (%)	383 (45.5)	274 (45.8)	86 (47.3)	23 (37.1)	
Openness, mean (SD)	0 (1)	-0.02 (0.99)	-0.01 (1.01)	0.21 (1.03)	0.214
Conscientiousness, mean (SD)	0 (1)	0.13 (0.97)	-0.28 (1.03)	-0.43 (0.89)	<0.001^a^^,^^b^
Extraversion, mean (SD)	0 (1)	-0.05 (0.98)	0.03 (1.08)	0.40 (0.89)	0.002^b^^,^^c^
Agreeableness, mean (SD)	0 (1)	0.01 (1)	-0.06 (1.01)	0.08 (1.02)	0.605
Emotional Instability, mean (SD)	0 (1)	-0.09 (0.98)	0.15 (0.99)	0.45 (1.06)	<0.001^a^^,^^b^
Mother’s education (N = 828) n (%)					0.049
Less than primary	48 (5.8)	32 (5.4)	11 (6.2)	5 (8.1)	
Primary	189 (22.8)	131 (22.2)	35 (19.8)	23 (37.1)	
Secondary	361 (43.6)	259 (44.0)	78 (44.1)	24 (38.7)	
University	230 (27.8)	167 (28.4)	53 (29.9)	10 (16.1)	
Father’s education (N = 800) n (%)					
Less than primary	54(6.8)	33(5.8)	14(8.2)	7(11.7)	0.013
Primary	208 (26.0)	147 (25.8)	37 (21.6)	24 (40.0)	
Secondary	388 (48.5)	282 (49.6)	81 (47.4)	25 (41.7)	
University	150 (18.8%)	107 (18.8)	39 (22.8)	4 (6.7)	

*Note*. Personality traits are given in *z*-scores.

*ANOVA and chi-Square Test.

^a^ *p* <0.05 between never smokers and triers.

^b^ *p* < 0.05 between never smokers and smokers.

^c^ *p* < 0.05 between triers and smokers.

To examine the independent associations of adolescent personality and parental education to smoking behaviour among adolescents, we fitted a set of three stage multinomial logistic regression models (Table 2). Adjusting for the effects of age and gender, lower levels of conscientiousness and higher levels of extraversion and agreeableness were associated with higher risk of being classified as both ever smoker and regular smoker (Model 1). The associations were stronger for the regular smoker category. Furthermore, higher parental education was associated with regular smoking (Model 2). When personality and parental education were taken together, the associations remained significant and similar in magnitude to those observed before (Model 3)

**Table 2:** Odd Ratios (OR) and 95% Confidence Intervals (95% CI) for the models containing personality traits and parental education, predicting adolescent smoking status.

	Model 1^a^		Model 2^a^		Model 3^a^	
	Triers	Regular smokers	Triers	Regular smokers	Triers	Regular smokers
**Demographic**						
Age	2.16 (1.61–2.91)***	2.19 (1.34–3.52)**	2.12 (1.59–2.82)***	1.54 (0.98–2.43)	2.21 (1.64–2.99)***	1.94 (1.18–3.20)**
Male	1.01 (0.72–1.53)	0.69 (0.37- 1.28)	1.00 (0.71–1.40)	0.690 (0.40–1.20)	1.05 (0.72–1.53)	0.69 (0.36–1.29)
**Personality**						
Openness	1.02 (0.85–1.21)	1.19 (0.89–1.58)			1.00 (0.84–1.20)	1.25 (0.93–1.68)
Conscientiousness	0.57 (0.46–0.71)***	0.39 (0.27–0.55)***			0.57 (0.46–0.71)***	0.39 (0.27–0.56)***
Extraversion	1.38 (1.12–1.70)**	2.43 (1.67–3.55)***			1.37 (1.12–1.69)**	2.42 (1.65–3.55)***
Agreeableness	1.15 (0.93–1.43)	1.29 (0.92–1.80)			1.16 (0.93–1.44)	1.28 (0.91–1.80)
Em. Instability	1.33 (1.10–1.60)**	1.76 (1.31–2.35)***			1.32 (1.10–1.59)**	1.77 (1.32–2.38)***
**Parent. education**			1.12 (0.94–1.33)	0.70 (0.54–0.89)**	1.11 (0.93–1.33)	0.65 (0.50–0.86)**

*Note*. Em. Instability = Emotional Instability; Parent. education = Highest parental education

*p < 0.05

**p<0.01

*** *p* < 0.001; N = 842.

^a^Reference category for both “triers” and “regular smokers” is “never smoker”. All models controlled for the RCT group (control and intervention).

To test whether parental education moderated the associations between students’ personality traits and smoking status, we fit an additional multinomial logistic model. In this model (Table 3) none of the interactions between personality and parental education was statistically significant.

**Table 3:** Odd Ratios (95% Confidence Intervals) for the model including interactions between adolescent personality and parental education predicting adolescent smoking status.

	Model 4	
	Triers	Regular Smokers
	OR (95% CI)	OR (95%CI)
**Demographics**		
Age	2.24 (1.66–3.03)***	1.96 (1.18–3.27)**
Male vs. Female	1.05 (0.72–1.53)	0.68 (0.36–1.28)
**Personality**		
Openness	1.01 (0.85–1.22)	1.35 (0.99–1.84)
Conscientiousness	0.57 (0.46–0.70)***	0.36 (0.25–0.52)***
Extraversion	1.37 (1.12–1.69)**	2.26 (1.53–3.33)***
Agreeableness	1.15 (0.92–1.43)	1.46 (1.01–2.11)*
Emotional Instability	1.32 (1.10–1.59)**	1.74 (1.28–2.36)***
**Parental education**	1.09 (0.90–1.31)	0.72 (0.51–1.02)
**Interactions**		
Parental education x O	0.88 (0.77–1.06)	1.11 (0.84–1.45)
Parental education x C	0.95 (0.77–1.18)	0.93 (0.68–1.28)
Parental education x E	1.10 (0.90–1.36)	0.74 (0.51–1.06)
Parental education x A	0.96 (0.77–1.19)	1.38 (1.00–1.89)
Parental education x EI	0.93 (0.78–1.13)	0.90 (0.67–1.21)

*Note*. OR: Odds Ratio; CI: Confidence Interval; O = Openness; C = Conscientiousness; E = Extraversion; A = Agreeableness, E.I. = Emotional Instability. *N* = 842.

*p < 0.05

**p < 0.01

***p < 0.001.

## Discussion

Our results indicate that parental education and adolescents’ personality traits are independently associated with adolescent smoking behaviour. Adolescents with higher levels of extraversion and neuroticism and lower level of conscientiousness were more likely to smoke. Furthermore, adolescents whose parents had lower educational level were more likely to smoke regularly. Finally, we found no evidence that parental education moderates the associations between adolescent’s personality and smoking behaviour.

Our results regarding extraversion and neuroticism corroborate those reported in previous studies conducted on adolescent samples [34, 35]. Of the five personality traits, neuroticism has been most consistently associated with smoking. One possible explanation is that adolescents with higher levels of neuroticism use smoking to reduce unpleasant emotions [35]. On the other hand, it is possible that individuals higher in extraversion show higher probability to smoke because both their high sociability and their increased dopaminergic activity that would be associated with increased likelihood of smoking [36]. Lastly, with respect to personality traits, we found that higher levels of conscientiousness were associated with lower likelihood of smoking in adolescence. This is not surprising, given that conscientiousness is most commonly associated with better health-related behavioural patterns in adult life, including lower prevalence of smoking [36–38].

Furthermore, our results are similar to those reported in previous studies that found an association between family SES and smoking among adolescents [12, 17]. We found an association between an indicator of family SES, highest parental educational level, and regular smoking, but we did not find an association between parental education and triers. It is possible that individual factors, such as personality, explain whether or not adolescents initiate smoking, while environmental factors, such as SES, contribute to explain why smoking eventually becomes a persistent habit. One proposed mechanism of the associations between parental affluence and adolescent health behaviours is through modelling processes [17]. To test this possibility, we utilised information on parental smoking collected two years prior to the data presented here. There were significantly more parent smokers among those with lower education (primary or less) than those of medium and higher education (36% vs 25% of smokers for mothers, and 37% vs 30% for fathers, data available upon request). Interestingly, parental education was also related to adolescent smoking when controlling for parental smoking (OR = 0.7; 95%CI 0.5–0.9, data available upon request) that could indicate also some effect of parental education independent of modelling behaviour.

Finally, parental education was associated with adolescent smoking independently of adolescent personality. This finding replicates and expands those reported by Chapman et al. [25] who utilised measures of own education and smoking behaviour in adults. Therefore, the two sets of predictors may act independently in adolescent as well as in adult populations. Future studies should also examine whether similar patterns apply to other substances [39].

This study had several limitations. First, the current analysis was limited to cross-sectional data. Although personality traits are relatively stable across the life span [40], longitudinal design is needed to better address causal direction of the effect. Future studies of smoking behaviour in adolescence should include a measure of personality traits prior to smoking initiation. Second, we include a measure of parental education as the only SES indicator. It is possible that a more comprehensive measure of parental SES would yield different results, especially with respect to interactions with personality traits [41]. Finally, we relied on self-reported measures of smoking behaviour. However, while direct methods such as coximetry exist, they suffer from reliability issues due to uneven inhalation and false positives due to exposure to environmental smoke. Coximetry was also not shown to accurately assess smoking in adolescents [42].

Our study contributes to the evidence that personality factors and parental education are important factors associated with smoking behaviour during adolescence. Furthermore, these factors should be addressed independently. Effective interventions tailored for individual personality traits and SES backgrounds could be useful to reduce future social disparities in morbidity and mortality.
